# Fog-basking behaviour and water collection efficiency in Namib Desert Darkling beetles

**DOI:** 10.1186/1742-9994-7-23

**Published:** 2010-07-16

**Authors:** Thomas Nørgaard, Marie Dacke

**Affiliations:** 1 Department of Biology. University of Lund. Sölvegatan 35, S-22362 Lund, Sweden

## Abstract

**Background:**

In the Namib Desert fog represents an alternative water source. This is utilised by Darkling beetles (Tenebrionidae) that employ different strategies for obtaining the fog water. Some dig trenches in the sand, while others use their own bodies as fog collectors assuming a characteristic fog-basking stance. Two beetle species from the genus *Onymacris *have been observed to fog-bask on the ridges of the sand dunes. These beetles all have smooth elytra surfaces, while another species with elytra covered in bumps is reported to have specialised adaptations facilitating water capture by fog-basking. To resolve if these other beetles also fog-bask, and if an elytra covered in bumps is a more efficient fog water collector than a smooth one, we examined four Namib Desert beetles; the smooth *Onymacris unguicularis *and *O. laeviceps *and the bumpy *Stenocara gracilipes *and *Physasterna cribripes*. Here we describe the beetles' fog-basking behaviour, the details of their elytra structures, and determine how efficient their dorsal surface areas are at harvesting water from fog.

**Results:**

The beetles differ greatly in size. The largest *P. cribripes *has a dorsal surface area that is 1.39, 1.56, and 2.52 times larger than *O. unguicularis*, *O. laeviceps*, and *S. gracilipes*, respectively. In accordance with earlier reports, we found that the second largest *O. unguicularis *is the only one of the four beetles that assumes the head standing fog-basking behaviour, and that fog is necessary to trigger this behaviour. No differences were seen in the absolute amounts of fog water collected on the dorsal surface areas of the different beetles. However, data corrected according to the sizes of the beetles revealed differences. The better fog water harvesters were *S. gracilipes *and *O. unguicularis *while the large *P. cribripes *was the poorest. Examination of the elytra microstructures showed clear structural differences, but the elytra of all beetles were found to be completely hydrophobic.

**Conclusions:**

The differences in fog water harvesting efficiency by the dorsal surface areas of beetles with very different elytra surface structures were minor. We therefore conclude that the fog-basking behaviour itself is a more important factor than structural adaptations when *O. unguicularis *collect water from fog.

## Background

The cold Benguela current runs along the South West African coast, creating one of the most arid habitats on earth; the Namib Desert [1]. Water is essential to all living organisms and this harsh environment presents a major challenge for all life forms. However, the cold coastal current not only suppresses rainfall over the desert, but is also the origin of fog that can reach as much as 100 km inland from the coast [2]. Fog brings water in the form of minute droplets that can deposit up to a litre of water per square metre on the mesh of an artificial fog screen during a day in the Namib Desert [3]. These fog events occur approximately 30 days per year in the inland desert [4], and represent a predictable source of water for the Namib Desert organisms [2].

The Namib Desert has a remarkably high variety of Darkling beetles (Tenebrionidae) and a handful of them actively exploit fog for water intake [5,6]. Some of these construct sand trenches or ridges to catch the fog, while *Onymacris unguicularis *and *O. bicolor *instead utilise their own body surface as a fog water collector [7-9]. By adopting a head standing posture facing into the wind, the fog water collects on their elytra and runs down to their mouth, to be imbibed by the beetles. This unique behaviour is termed fog-basking [7]. The advantage of fog collection for water intake in the extremely arid desert is obvious, and becomes critical when rainfall is absent over prolonged periods of time. Long term studies on the population density of Darkling beetles in the Namib Desert clearly shows that the fog collecting beetles are still present in great numbers during periods of low rain fall, whereas the large majority of Darkling beetles that lack this adaptation disappear or decline to less than 1% of their mean abundance [5].

The mechanism by which fog water forms into large droplets on a beaded surface has been described from the study of the elytra of beetles from the genus *Stenocara *[10]. The structures behind this process are believed to be hydrophilic peaks surrounded by hydrophobic areas; water carried by the fog settles on the hydrophilic peaks of the smooth bumps on the elytra of the beetle and form fast-growing droplets that - once large enough to move against the wind - roll down towards the head. The application of this mechanism for water collection by fog-basking beetles has however been questioned [9]. Hamilton and co-authors do not challenge the mechanism presented for fog collection, but argue that the beetle studied to reveal this mechanism never actually actively collects fog water in nature. That fact that Hamilton and colleagues [9] also re-identify the model beetle in the study by Parker and Lawrence [10] as a *Physasterna cribripes *rather than a *Stenocara *sp. does not influence their main argument, as neither of these genera are represented among the few Darkling beetles so far known to actively collect water from fog [5]. A quick inspection of the two beetles that are well known to fog-bask - *O. bicolor *and *O. unguicularis *- reveals that the elytra of these beetle do not carry the numerous bumps that were described as part of the water collecting mechanism in *Stenocara *(or possibly *Physasterna*), but rather have smooth elytra surfaces with regular grooves.

The first aim of the present account is to identify and describe a possible fog-basking behaviour in four desert beetles (including *Onymacris*, *Stenocara *and *Physasterna sp*.) in a temperature controlled indoor fog chamber. Irrespective of whether these beetles have evolved a fog-basking behaviour or not, elytra covered in bumps and valleys could still be a better collectors of fog water than the smooth surface with grooves described for the fog-basking beetles. In the second part of this study, we compare the water collecting efficiency of these two types of structurally different dorsal surfaces.

## Methods

### Beetles

**Figure 1:**
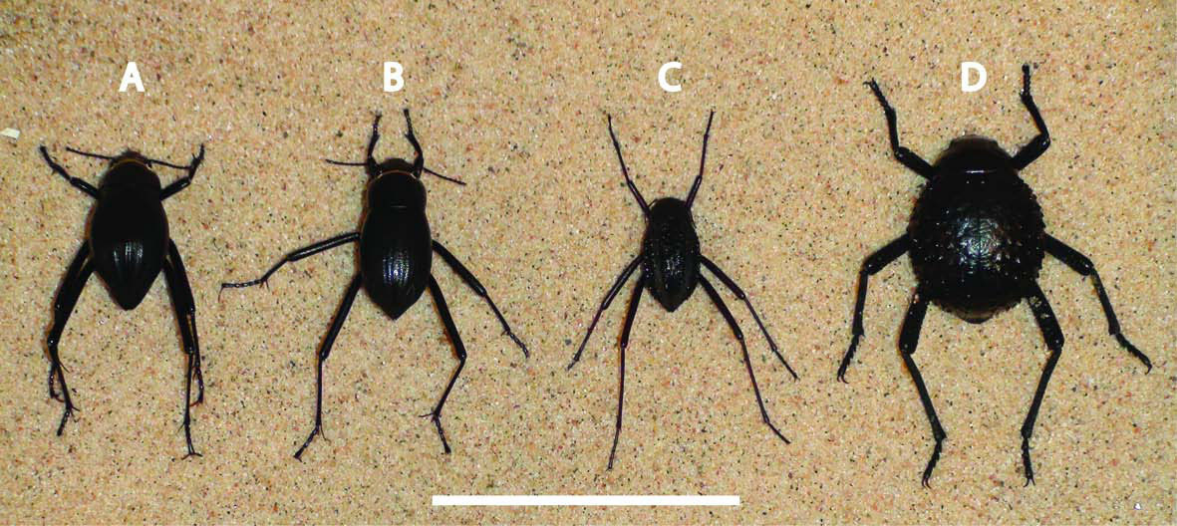
**Size difference between the four model beetles**. Examples of specimens from each beetle species placed next to each other for size comparison. A: *O. unguicularis*, B: *O. laeviceps*, C: *S. gracilipes*, and D: *P. cribripes*. The dorsal surface area of *P. cribripes *was found to be 1.39 times larger than *O. unguicularis*, 1.56 times larger than *O. laeviceps*, and 2.52 times larger than *S. gracilipes*. Scale bar: 3 cm. See Methods for how size difference was determined.

The fog collecting behaviour of four tenebrionid beetle species was compared: *Onymacris unguicularis *(Figure 1A) is known to fog bask and has a smooth dorsal surface with wide grooves [7]. *Onymacris laeviceps *(Figure 1B) has a similar surface structure, albeit with finer grooves, and inhabits the same sand dune habitat as *O. unguicularis*. It is nevertheless, not known to fog-bask but does drink from fog-dampened surfaces [5]. *Stenocara gracilipes *(Figure 1C) and *Physterna cribripes *(Figure 1D) are found outside the sand dune habitat and have elytra with a more or less regular array of smooth bumps. It is a matter of debate if either of these two species or genera fog-bask or not [5,9,10]. *Onymacris unguicularis *and *O. laeviceps *were collected in the sand dunes just south of the Kuiseb River (S 23 20.993; E 14 47.387). *Stenocara gracilipes *were collected in a rocky side canyon to the Kuiseb River canyon (S 23 18.011; E 15 45.533), and *P. cribripes *were collected on the gravel plains around the Gobabeb Training and Research Centre (S 23 33.713; E15 02.461).

### Elytra surface structures

The differences in the microstructures of the beetles' elytra were examined using a dissecting microscope and Scanning Electron Microscopy (SEM). Animals used for SEM were air-dried and sputter coated with gold-palladium (40/60). Sudan III staining was applied to test for wax, i.e. hydrophobic properties of the beetles' elytra. Whole beetles were carefully placed in 50% alcohol for three minutes, and then in a Sudan III solution for another three minutes. Following this the beetles were rinsed first with 70% alcohol and then distilled water. In order to minimise the risk of erroneous results due to transport and storage this procedure was carried out at the Gobabeb Training and Research Centre within three hours of the beetles' capture.

### Experimental set-up and identification of fog-basking behaviour

Studies of fog-basking behaviour and fog water collecting efficiency were conducted under laboratory conditions at Lund University, Sweden. The four beetle species were kept together in sand-filled boxes under the same living conditions (12 h light:12 h dark, 24°C, and 42-45% relative humidity). The animals were given water ad libitum at the time of export from Namibia and again a week after arriving in Sweden. Experiments were conducted two weeks after the animals last had received water.

The conditions of a Namib Desert dune during a fog event were emulated using a fog producing machine (Super fog, Lucky reptile, Waldkirch, Germany) placed in a 50 L refrigerator (Waves wc-16007). The fog producing machine generated fog travelling less than 0.1 m/s and produced 325 ml fog water per hour. The "fog chamber" was equipped with a glass door to allow observations and video recordings of the beetles inside, and the temperature was kept within a relevant range between 10 to 15°C and a relative humidity (RH) above 97% [9,11]. The microclimate in the fog chamber was measured with a miniature weather station (Silva ADC PRO, Silva Sweden AB). Live beetles were studied one by one in a square arena (17 × 17 cm) with sand shaped into a small ridge oriented perpendicular to the direction of the fog.

The time from when the beetle was placed in the chamber until it assumed a fog-basking position was recorded. This was defined as the time at which the beetle had oriented itself with the back towards the fog and assumed a static position with its head tilted downwards for a minimum of 2 min. The angle at which the fog-basking beetle positions itself during the water-collecting head stand was determined from a photograph taken directly from the side of the fog-basking beetle and measured as the angle between horizontal and the flat under side of the beetle. If no head stand or other fog-basking behaviour could be observed, the beetle was removed from the chamber after 20 min and was excluded from the following behavioural tests.

In a second set of experiments, the fog chamber was either heated up to 20-23°, or kept cold without any fog. This made it possible to determine what the triggering factor is behind the characteristic head standing behaviour of the fog-basking beetles. The beetles were again placed in the chamber one by one, and the time until the beetles assumed the characteristic head stand was recorded under the two different chamber conditions; warm and damp or cold and dry. The beetles were removed from the set-up after 20 min.

### Quantification of water collection

Prior to the third experiment, the beetles were killed by freezing, had their legs and antennae removed and were positioned on a stand by the use of model clay. The ventral sides of the beetles were positioned in the angle previously determined from live beetles fog-basking in the fog chamber (see above). An Eppendorf tube for water collection was placed under the apex of each beetle's head. The beetles were then placed in the fog chamber in array of 16. The array was made up of four independent rows into which one specimen from each species was placed in a random position. After two hours in the fog chamber, the water collecting efficiency of each beetle in the array was established as the weight difference of the Eppendorf tube before and after the treatment. The experiment was repeated 5 times, using a total of 20 beetles from each species.

After each treatment, coloured latex was applied to the head, pronotum, and elytra of the beetles to establish the relative sizes of the beetles' dorsal surface areas. The latex casts were placed on a white background, flattened under a glass plate and photographed. The relative dorsal surface area of each beetle was determined from the number of coloured pixels from the standardised photographs of the flattened casts, and normalised to the largest beetle. This supplied us with a conversion factor to obtain the relative amount of water captured per area, rather than the total amount of water captured for each beetle species.

## Results

### Elytra surface structures

**Figure 2:**
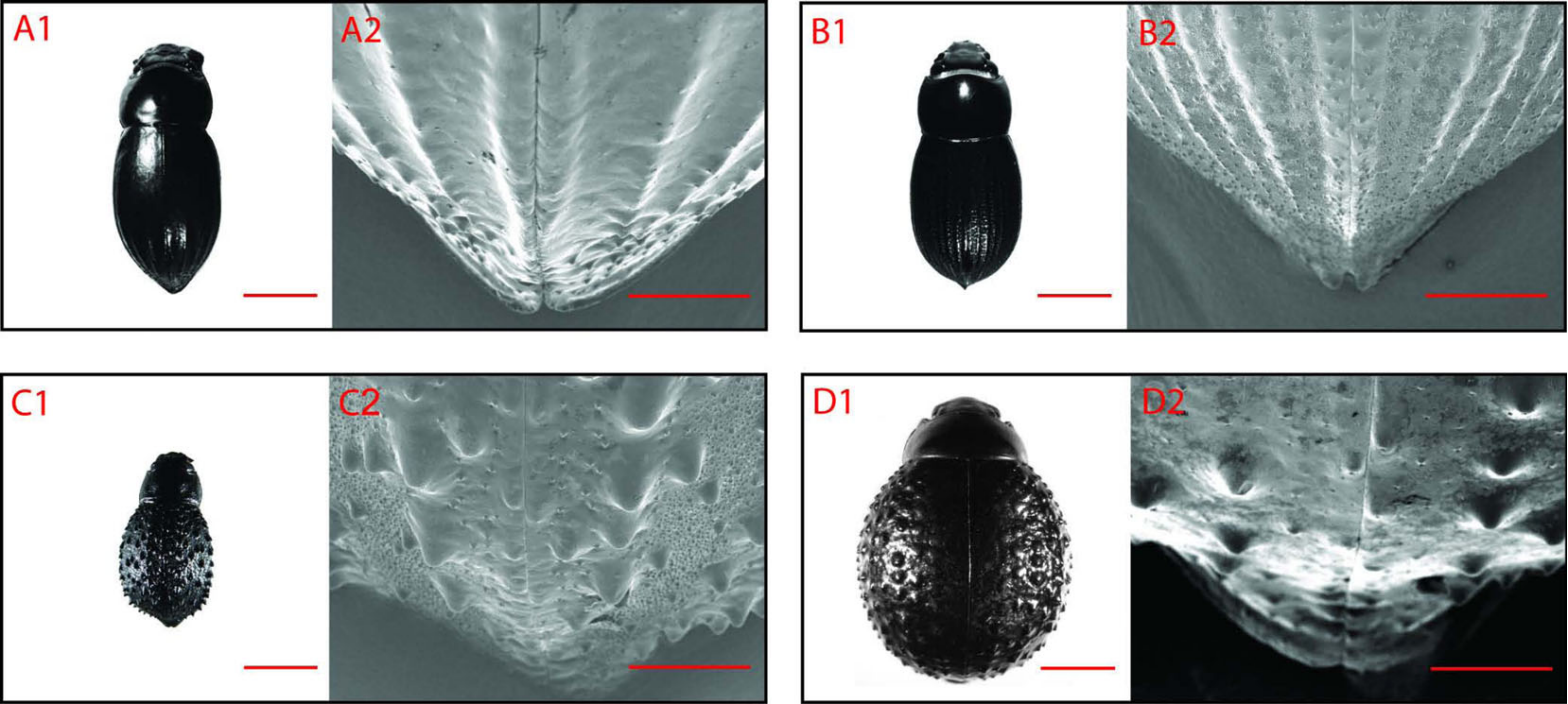
**Elytra structures**. A) *Onymacris unguicularis*, B) *Onymacris laeviceps*, C) *Stenocara gracilipes*, and D) *Physasterna cribripes*. A1-D1) Extended Depth Focus images of examples of the experimental animals obtained with a dissection microscope. Scale bar = 5 mm. A2-D2) Scanning Electron Microscope images of the apex of the elytra. Scale bar = 1 mm.

SEM images and photos taken through a dissection microscope show details of the pronounced differences in elytra structure among the four beetle species (Figure 2). Whereas the pronotum on all beetles is rather smooth, it is the elytra that have different structures. The elytra of *O. unguicularis *are almost completely smooth (Figures 2A1) except for the posterior half that has large distinct grooves, approximately 0.5 mm wide, divided by narrow ridges (Figures 2A2). The elytra of *O. laeviceps *have much finer grooves (Figures 2B1), approximately 0.1 mm wide (Figures 2B2), that cover almost the entire elytra. The valleys of the fine grooves are not as smooth as those of *O. unguicularis *but rather have a coarser surface. In live animals, the posterior half of *O. laeviceps *has a blue-gray colouration (Figures 2B1,2). The elytra of the small *S. gracilipes *are covered in jagged bumps that form irregular lines (Figures 2C1), although there are also bumps in between the lines (Figures 2C2). The elytra of the large *P. cribripes *likewise have bumps that form irregular rows with additional bumps in between (Figures 2D1). The bumps are slightly rounder than those of *S. gracilipes *(Figures 2D2) and are found over the entire elytra, with a smooth stripe on either side of the suture of the beetles' fused elytra.

**Figure 3:**
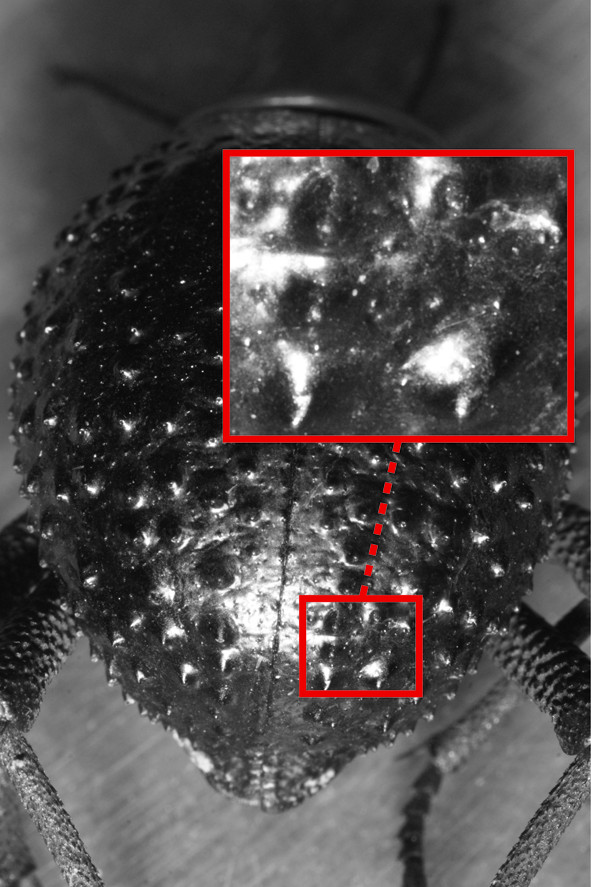
**Hydrophobic dorsal surface of *Physasterna cribripes***. An example of a *P. cribripes *treated with Sudan III staining in order to examine if the beetles have hydrophilic zones that could facilitate the collection of water from fog. The Sudan III staining make wax covered i.e. hydrophobic areas shiny. The magnification shows the shining hydrophobic peaks of the bumps on the elytra.

To test for possible hydrophilic properties of the elytra, the beetles were treated with Sudan III. This procedure gives wax covered, i.e. hydrophobic, areas on the beetles an orange coloured shine that is distinctly different to the normal black colour of the elytra. Hydrophilic areas will therefore remain black and can easily be identified with this treatment. However, no hydrophilic areas could be identified either on the elytra, pronotum or head in any of our four model species including *P. cribripes *(Figure 3).

### Fog-basking behaviour

**Figure 4:**
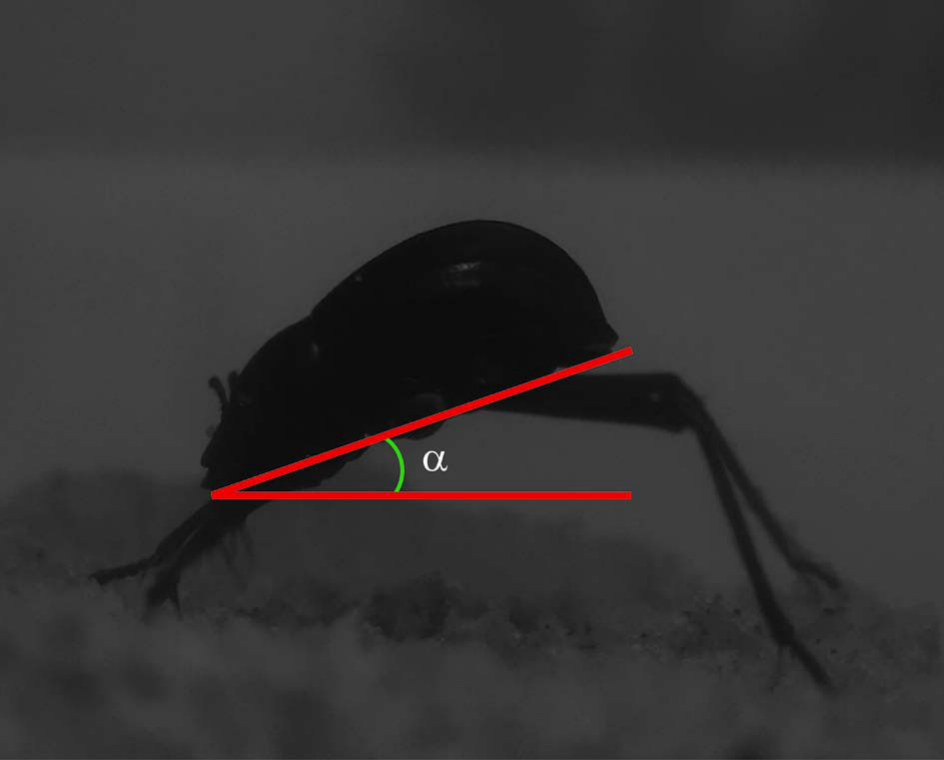
**Fog basking posture of *Onymacris unguicularis***. Photograph of a fog-basking *O. unguicularis *inside the fog chamber (see methods) exhibiting a characteristic fog-basking head stand. This posture allows fog water collected on the beetle's dorsal surface to trickle down to its mouth.

Out of the four species of beetle tested in the fog chamber under conditions imitating those in the Namib Desert during a fog event, only *O. unguicularis *could be observed to actively collect water from the fog. These beetles (N = 15) positioned themselves on the top of the sand ridge in the chamber and assumed a fog-basking position after 114.5 ± 9.28 sec. (mean ± SE). The starting point of this behaviour was defined as the time at which *O. unguicularis *had oriented itself with the back towards the fog and thereafter remained in this static position with its head tilted downwards for a minimum of 2 min. The ventral side of the beetle was held at an angle of approximately 23° (22.7 ± 0.65°, mean ± SE, N = 15) to horizontal during these events (Figure 4). In contrast, the other three beetle species kept walking around in the arena during the 20 minutes they were observed in the fog chamber (*O. laeviceps*: N = 16, *P. cribripes*: N = 16, and *S. gracilipes*: N = 14). These three species were consequently excluded from further behavioural experiments in the fog chamber.

The fog-basking *O. unguicularis *(N = 12) was again tested in the fog chamber at temperatures equivalent to what exists under natural fog events (11.9 ± 0.23°C, mean ± SE), but this time without any fog in the chamber (25.5 ± 0.34% RH, mean ± SE). With no fog present, *O. unguicularis *did not display any fog-basking behaviour during the 20 minutes they were observed in the chamber. However, if the temperature was elevated to room temperature (21.9 ± 0.26°C, mean ± SE, N = 12) and the chamber was filled with fog, six out of twelve beetles assume a fog-basking position after 175 ± 21.65 sec. (mean ± SE, N = 6). The other six beetles remained active and moved around for the 20 minutes they were observed, but never adopted a static head standing position. High humidity, rather than low temperature, is thus the critical condition under which the fog-basking beetles will assume their characteristic head stand for water collection. However, a combination of fog and low temperatures is the strongest trigger for this behaviour.

### Fog-water collection efficiency

**Figure 5:**
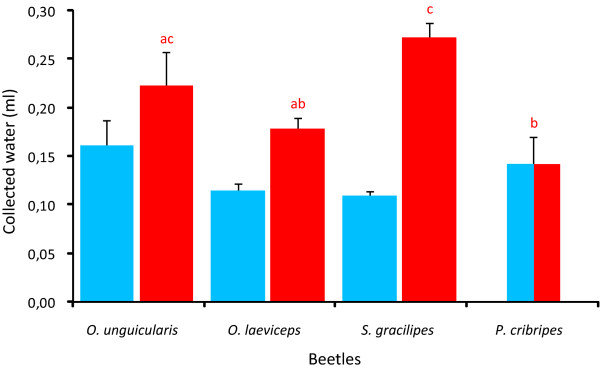
**Fog harvesting efficiency**. Beetles killed by freezing had their legs and antennae removed and were positioned head down at an angle of 23° in a fog chamber. An Eppendorf tube for water collection was placed under each beetle's head. After two hours in the chamber the total amount of water captured by each of the four beetle species was measured (blue). The relative dorsal surface area of each beetle was determined and normalized to the largest beetle. This conversion factor was used to obtain the relative amount of water captured per dorsal surface area (red). The columns show mean ± SE. Columns marked with matching lower-case letters above are not significantly different at p < 0.05 (Kruskal-Wallis test and Dunn's Multiple Comparisons Test).

Irrespective of their ability to actively collect water from fog in the fog chamber or not, the ability of the four beetle species to passively collect water from fog was tested from dead specimens (20 of each species) mounted head down at an angle of approximately 23° (as previously determined from fog-basking in the fog chamber, see above). After two hours in the fog chamber, *Onymacris unguicularis *and *O. laeviceps*, that have smooth elytra with grooves had collected 0.16 ± 0.03 (mean ± SE) and 0.11 ± 0.01 ml of water respectively. *Stenocara gracilipes *and *P. cribripes*, that have elytra with an array of bumps had, during the same time, collected 0.11 ± 0.01 ml and 0.14 ± 0.03 ml respectively. *Onymacris unguicularis *and *P. cribripes *showed a tendency to harvest more fog water than *O. laeviceps *and *S. gracilipes*, but not significantly so (Kruskal-Wallis Test, p = 0.26) (Figure 5). Despite distinctly different elytra structures (and behaviours) the four beetles collected the same amount of water over a 2 hour period in the fog chamber. The four beetle species do, however, vary in size (Figure 1), and to assess the water harvesting efficiency of the different surface structures we also have to take the size of the beetles into account.

The relative sizes of beetles' dorsal surface area (the dorsal part of the head, the pronotum, and the elytra) were established from coloured latex casts of the different beetles used in the water collection efficiency experiments. We found that the dorsal surface area of the large *P. cribripes *on average is 1.39 times larger than the same region in *O. unguicularis*, 1.56 times larger than *O. laeviceps*, and 2.52 times larger than that of the smallest beetle *S. gracilipes*. By applying these relative differences in dorsal surface areas as conversion factors to the absolute amount of water collected per species, we get an estimate of the water collecting efficiency of each species that is independent of their sizes.

Despite the fact that *O. unguicularis *is the only beetle in this study that actively collects water from fog, it does not seem to come equipped with any surface structures that are superior for this purpose compared to those of the other beetles. In fact, no significant difference in water harvesting per unit of dorsal surface area can be found between *O. unguicularis *(0.22 ± 0.04 ml, mean ± SE, N = 20) and *O. laeviceps *(0.18 ± 0.01 ml), or *O. unguicularis *and *S. gracilipes *(0.27 ± 0.02 ml), (Kruskal-Wallis test, p < 0.0001, Dunn's Multiple Comparisons Test, p > 0.05, in both cases). The water collecting efficiency of the big *P. cribripes *(0.14 ± 0.03 ml) is, however, significantly lower than that of the fog-basking *O. unguicularis *(Dunn's Multiple Comparisons Test, p < 0.05). The small (*S. gracilipes*) and the big (*P. cribripes*) both have elytra with distinct bumps, but the water collecting efficiency of these two beetles come out in the high and low end of the spectrum, respectively, with a significant difference between the two (Dunn's Multiple Comparisons Test, p < 0.05). In fact, *S. gracilipes *harvests almost twice as much water per surface area unit (0.27 ± 0.02 ml) during the two hours in the fog chamber compared to *P. cribripes *(0.14 ± 0.03 ml).

## Discussion

### Fog-basking behaviour in a fog chamber

In this study, four Darkling beetles from the Namib Desert were exposed to fog in a small fog chamber. The temperature in the chamber was set at 10-12°, which is a temperature range similar to that of a fog event in Namib Desert [9,11]. When placed in a sand arena in the chamber, the fog-basking beetle *O. unguicularis *readily assumed their characteristic fog-basking stance [5,6] after a little more than 2 minutes in the chamber. The static head stance assumed by *O. unguicularis *while fog-basking in the chamber (Figures 4) was very similar to that documented for the same species while fog-basking at the crest of a sand dune during a fog event in the Namib Desert [7]. The other three beetles (*O. laeviceps, S. gracilipes *or *P. cribripes*) remained active but did, at no time, assume a similar stance during their 20 minutes in the fog chamber. The lack of a fog-basking behaviour in these three species of beetles is in accordance with long term observations of Darkling beetles in the Namib Desert, [5], where only two out of approximately 200 beetle species inhabiting this area have ever been observed to fog-bask - both from the genus *Onymacris*.

Fog-basking has also been reported within the genus *Stenocara *[10], but any comparison to our results from the fog chamber, is complicated by the fact that the identification of the beetle has been questioned [9]. Hamilton and co-authors identify the beetle as a *P. cribripes *(Figures 2D1 and 2D2), and a comparison between our model beetles and the illustration in the paper by Parker and Lawrence [10] supports this re-identification. For clarity, we will from here on treat the beetle from the study on water capture by a desert beetle by Parker and Lawrence [10] as a *P. cribripes*.

From our study in the fog chamber we could not reproduce the forward tilt into the wind that has been reported for *P. cribripes *[10]. This difference in behaviour could be a consequence of suboptimal conditions in the fog chamber, but the fact that *O. unguicularis *readily and predictably fog-basks in the same artificial environment supports the validity of the experimental setup. However, *P. cribripes *and many other tenebrionid beetles will also assume a tilting posture as a common alarm response [9]. The beetle then sticks its head into the ground, spreads its legs wide, and raises the rear part of its body. This posture resembles fog-basking and could have been mistaken for it in the study by Parker and Lawrence [10].

Fog was found to be the triggering factor for *O. unguicularis *to assume the fog-basking stance. None out of twelve beetles assumed this stance at low temperatures with no fog, but half of the tested *O. unguicularis *engaged in fog-basking when exposed to fog at approximately 23°C. In contrast, all *O*. unguicularis placed in a chamber filled with fog at temperatures similar to those under a natural fog event in the Namib Desert [9] assumed a fog-basking stance. This indicates that the temperature is a contributing, but not critical factor, for eliciting this behaviour.

The recorded tolerance for variability in the factors that trigger fog collection further supports our finding that other beetle species do not engage in this behaviour. Even if the temperature in the chamber might not have been set at the absolute critical temperature to elicit fog-basking behaviour in *O. laeviceps, S. gracilipes *or *P. cribripes*, we would still expect a few of them to assume a fog-basking stance when placed in the fog chamber. This was never observed.

### Water capturing efficiency by beetle elytra

Dead specimens of *O. unguicularis, O. laeviceps, S. gracilipes *or *P. cribripes were *placed in a fog-basking position. The beetles were oriented head down at an angle of 23°, as measured from the fog basking *O. unguicularis*. Exposing the dead specimens to fog for two hours under low temperature in the fog chamber did not reveal any significant differences in the total amount of water captured between the species. The beetles, however, differ greatly in size (Figure 1). The water capture was therefore adjusted for the difference in surface area by normalising the data to the surface area of the largest beetle *P. cribripes*. The water capture corrected for beetle size showed significant differences in water capturing efficiency, with *O. unguicularis *and *S. gracilipes *being the better fog water harvesters. These results reveal that the small beetle *S. gracilipes *is as efficient a fog water harvester, when measured per square unit of dorsal surface, as the bigger *O. unguicularis*, even though it never has been observed to actively fog-bask in nature [5] or in our fog chamber.

The high water collecting efficiency recorded for *S. gracilipes *is most likely a result of its relatively smaller size. Other organisms in the Namib Desert use fog as an important source of water, and small leaves have recently been shown to be an important factor for plants when harvesting water from fog [12]. This is because small or narrow leaves have only thin boundary layers (an envelope of slow moving air around the object) that allow the fog water to collect on the surface of the leaf, rather than being blown around the leaf and away [13,14]. Also, a smaller beetle should have a thinner boundary layer and would thus be better at collecting water from the fog laden wind. In the light of this, it is less surprising that the small *S. gracilipes *proves to be a good fog-water harvester as measured per unit area, and the big *P. cribripes *the worst. Interestingly, the 1.81 times larger *O. unguicularis *is as good at fog-harvesting as *S. gracilipes*, but not the slightly smaller *O. laeviceps*. This indicates that *O. unguicularis - *in addition to their fog-basking behaviour - could have structural adaptations on their elytra to improve water harvesting from fog. Part of this favourable outcome for *O. unguicularis *could of course be influenced by the fact that all beetle species were mounted in the fog-basking position assumed by live *O. unguicularis*. The finding that *P. cribripes *turns out to be the worst water harvester of all four beetles, despite its reported hydrophobic and hydrophilic elytra structures for droplet formation [10] does, however, warrant a comparison between the highly different elytra structures of *O. unguicularis *and *P. cribripes *(Figures 2A1 and 2D1).

### Elytra structures of *Onymacris unguicularis *and *Physasterna cribripes*

On a macroscopic scale, the elytra of *P. cribripes *are covered in an array of bumps, 0.5-1.5 mm apart, each about 0.5-1.5 mm in diameter (Figures 2D1). This is in accordance with earlier reports on the elytra of this beetle [10] (for species identification see above). The fog-basking *O. unguicularis *rather have smooth elytra that, in the back half, are folded into regular grooves that bend towards the apex of the body. The grooves are approximately 0.5 mm wide and approximately 0.1 mm apart. These bumps and grooves could, theoretically, form the basis of the combination of hydrophilic and hydrophobic points that have been shown to improve water capture from fog [10]. The Sudan III staining, however, did not reveal any hydrophilic areas on the elytra of any of the beetles (Figure 3). This observation does not agree with earlier reports of *P. cribripes *having hydrophilic zones on the apex of their elytra bumps [10]. In the former study of this beetle, the elytra were treated with Red O. This is a different staining method that also tests for the presence of waxes, but details on the conditions that the beetles were kept under prior to staining are unfortunately not given. In the present study, the beetles were stained within three hours of capture to avoid any artificially induced wear on the cuticle of the beetles from stress and/or the exposure to a substrate different from that of their natural environment. The apex of the peaks would be especially exposed to such wear, but without any further information on the pre-staining procedures by Parker and Lawrence [10] it is hard to draw any further conclusions from the diverging results. Our results do, however, indicate that water harvesting in the fog-basking beetle *O. unguicularis *is not improved by a combination of hydrophilic and hydrophobic points on its elytra. If the comparatively high fog-harvesting efficiency on the smooth surface of *O. unguicularis *is caused by structural adaptations the effect of these appears to be small. The observation that *S. gracilipes - *which is covered in bumps, rather than grooves - is an equally efficient harvester of water if placed in a fog-basking position, further suggests that a combination of grooves and smooth surfaces are in no way critical for fog-harvesting in the darkling beetles. We therefore conclude that water harvesting from fog in the Namib Desert beetle *O. unguicularis *is primarily a consequence of behavioural, rather than structural adaptations to the utilisation of an alternative source of water in an environment where rain is a rare event.

## Conclusions

In accordance with earlier reports from the field, we find that *O. unguicularis *is the only one of our four model beetles that assumes a head standing fog-basking stance in a low temperature environment with artificially produced fog. A comparison of the fog-water harvesting efficiency of the elytra of the fog-basking and non-fog basking beetles reveals that the small *S. gracilipes *and the fog-basking *O. unguicularis *were the better fog water harvesters, while the large *P. cribripes *was the worst. The differences in water collecting efficiency were however minor and we conclude that it is the fog-basking behaviour itself (i.e. moving to the top of the sand dune ridges and assuming the fog-basking stance) rather than physical adaptations that is the important factor allowing *O. unguicularis *to exploit fog as an alternative source of water in the extremely arid Namib Desert.

## Competing interests

The authors declare that they have no competing interests.

## Authors' contributions

TN had the initial idea, performed the experiments and analysed data. TN and MD designed the experiments and interpreted the data. Both authors wrote the manuscript and both authors read and approved the final manuscript.
